# Casual effects of gut microbiota on risk of infections: a two-sample Mendelian randomization study

**DOI:** 10.3389/fmicb.2023.1284723

**Published:** 2023-10-10

**Authors:** Beibei Lyu, Jinghang Ma, Yongyu Bai, Zhen Feng

**Affiliations:** ^1^School of the First Clinical Medical Sciences (School of Information and Engineering), Wenzhou Medical University, Wenzhou, China; ^2^Department of Gastrointestinal Surgery, The First Affiliated Hospital of Wenzhou Medical University, Wenzhou, China

**Keywords:** Mendelian randomization, gut microbiota, infection susceptibility, genetic relationships, genome-wide association study

## Abstract

**Background:**

The correlation between gut microbiota and infections has garnered significant attention in previous studies; nevertheless, our understanding of the causal relationships and mechanisms between specific microbial species and infections remains limited.

**Methods:**

This study aimed to employ Mendelian randomization (MR) using single-nucleotide polymorphisms (SNPs) and genome-wide association study (GWAS) data of European ancestry to explore the genetic-level relationships between distinct types of gut microbiota and susceptibility to infections. Our analysis encompassed three prevalent infections: intestinal infections, pneumonia, and urinary tract infections, while concurrently examining various types of gut microbiota.

**Results:**

We identified 18 protective gut microbiotas alongside 13 associated with increased infection risk. Particularly noteworthy are certain microbial communities capable of producing butyrate, such as the *Ruminococcaceae* and *Lachnospiraceae* families, which exhibited both favorable and unfavorable effects. Additionally, we observed a few certain communities linked to infection susceptibility, including *ErysipelotrichaceaeUCG003* (OR = 0.13, 95% CI: 0.054–0.33, *p* = 1.24E-05), *Collinsella* (OR = 3.25, 95% CI: 2.00–5.27, *p* = 1.87E-06), and *NB1n* (OR = 1.24, 95% CI: 1.09–1.40, *p* = 1.12E-03).

**Conclusion:**

This study reveals complex relationships between gut microbiota and various infections. Our findings could potentially offer new avenues for exploring prevention and treatment strategies for infectious diseases.

## 1. Introduction

Infections, such as intestinal infections, pneumonia, and urinary tract infections, pose significant risks to human health, often leading to immune system disorders and even fatal outcomes (Naghavi et al., 2017; National Vital Statistics Reports, 2020). The gut, acknowledged as a complex environment, engages in intricate interactions that substantially contribute to host homeostasis (Clark and Coopersmith, 2007). Concurrently, gut microbiotas are recognized for their pivotal role in governing physiological functions, with numerous studies illustrating their impact on host metabolism and disease progression (Clemente et al., 2012; Gao et al., 2023).

Previous researches have demonstrated that various infectious diseases can arise from disturbances or deficiencies in gut microbiota, while the gut microbiota itself can act as a protective mediator during infections (Schuijt et al., 2016; El-Sayed et al., 2021; Gagnon et al., 2023). However, the results have yet to provide a definitive picture due to the complexity of identifying specific microbiota types and their underlying mechanisms. Randomized controlled trials (RCTs) are widely acknowledged as robust study designs for establishing causal relationships. Nonetheless, logistical challenges and high costs can impede their implementation, and RCTs often offer qualitative rather than quantitative assessments of effects. Mendelian randomization (MR), employing single-nucleotide polymorphisms (SNPs) and genome-wide association studies (GWAS), presents a compelling method for elucidating causality and effect measurement (Emdin et al., 2017). By utilizing genetic variants as substitutes for adjustable elements, MR allows us to infer potential causative links between exposures and outcomes. Through leveraging natural genetic variations, this approach offers a quasi-randomized framework to tackle confounding issues in observational studies. MR has emerged as a valuable tool in epidemiology and genetics, facilitating the exploration of causality.

The aim of this study was to explore the genetic-level correlation between distinct categories of gut microbiota and susceptibility to infectious diseases. Our investigation encompassed three prevalent bacterial infections: intestinal infections, pneumonia, and urinary tract infections. In addition, we conducted a comprehensive analysis of various types of gut microbiotas. Simultaneously, multiple kinds of gut microbiotas were included in the assessment.

## 2. Materials and methods

### 2.1. Study design

**Figure 1:**
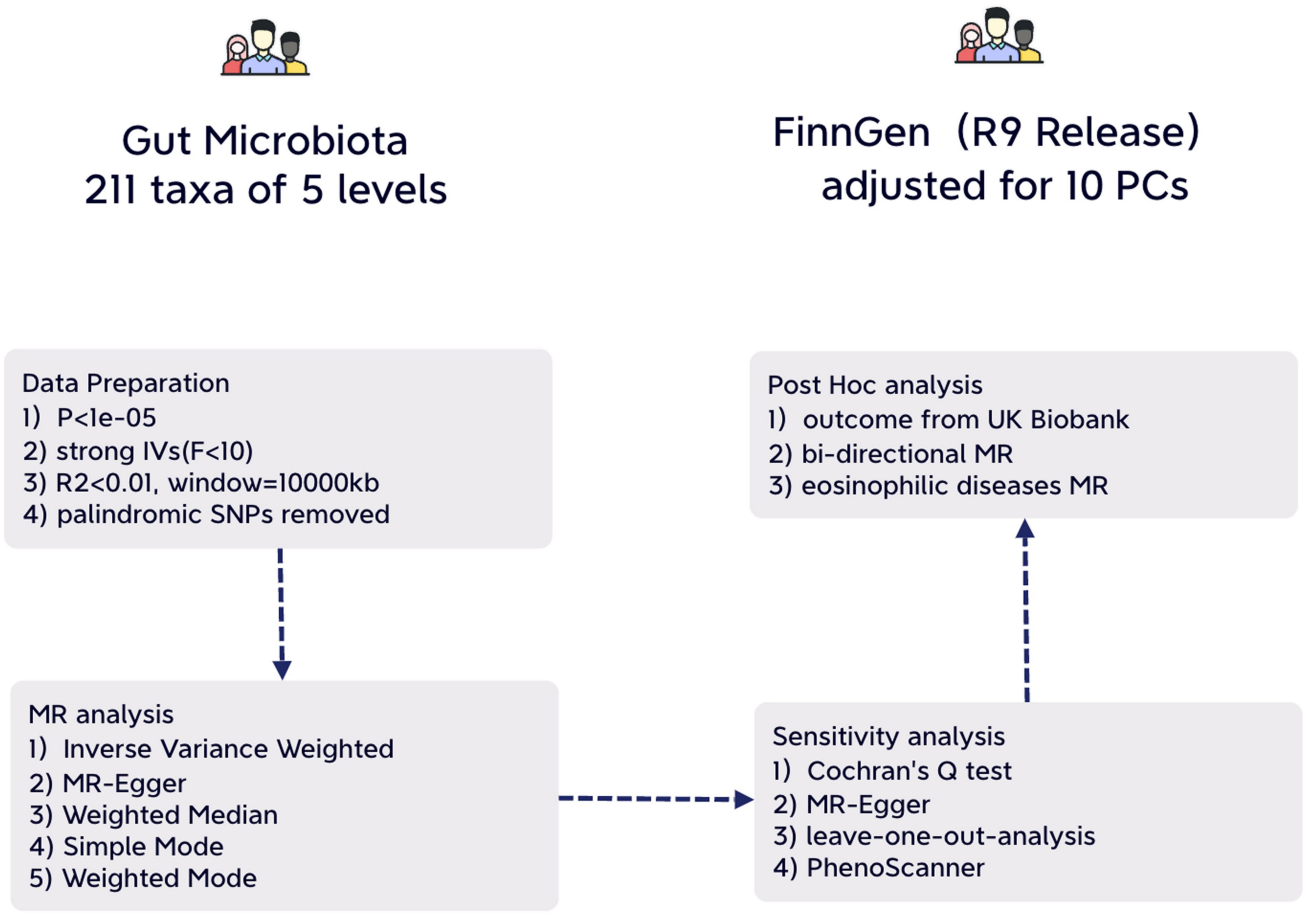
An illustrative overview depicting the structure of study design.

We adhered to the STROBE-MR guidelines for our study methodology (Additional File 4. Supplementary Table S1) (Skrivankova et al., 2021). Our approach involved a two-sample MR design using summary-level statistics derived from three distinct consortia. To minimize potential bias, both exposure and outcome data were limited to individuals of European ancestry (Sanderson et al., 2022). It’s important to note that all statistics utilized in this study are sourced from publicly available databases with participant consent and ethical approvals, rendering any further permission unnecessary for our study (Figure 1).

### 2.2. Instrumental variables (IVs)

The criteria for selecting IVs were as follows: (1) Only independent SNPs of gut microbiota were included in the analysis, with a clumping window size of 10,000 kb and an r2 threshold of less than 0.01. (2) To ensure an adequate number of SNPs for exposures, a threshold *p*-value of 1e-05 was applied (Li et al., 2022). (3) SNPs with F-statistics power below 10, indicative of potential weak instrumental bias, were excluded to mitigate this risk. (4) Additionally, palindromic SNPs were also excluded from consideration.

### 2.3. Data sources

Instrumental variables (IVs) for a total of 211 intestinal microflora (131genera, 35 families, 20 orders, 16 classes and 9 phyla) involved in this study were accessed from a genome-wide meta-analysis published on MibioGen consortium (Wang et al., 2018; Kurilshikov et al., 2021). There are 18,340 participants from 24 cohorts, mostly have European ancestry (n = 13,266), and 16S rRNA gene sequencing profiles were coordinated (Kurilshikov et al., 2021). Summary statistics from GWAS on infectious diseases were acquired from independent cohorts of European ancestry: UK biobank (Gagliano Taliun et al., 2020) and FinnGen Release (Kurki et al., 2023). The GWAS statistics from UK Biobank were modified for genetic relatedness, sex, birth year, and the first four principal components as covariates using SAIGE (Gagliano Taliun et al., 2020). The GWAS statistics from FinnGen (R9) were also adjusted *via* SAIGE, using sex, age, genotyping batch and ten PCs as covariates (Kurki et al., 2023). In these two database, cases and controls were defined based on ICD10-codes. For each infection, MR analysis were performed on data from two consortium respectively, mainly based on data from FinnGen.

### 2.4. Statistical analysis

The *F*-statistic power was estimated by using the formula: *F*= $\beta^{2}$ / $se^{2}\;$ (Wang S. et al., 2022). *F*-statistic power calculations were performed for all genetic variants related to exposures. We employed various methods, including Inverse Variance Weighted (IVW), MR Egger, Weighted Median, Simple Mode, and Weighted Mode, to analyze the association between gut microbiota and infectious diseases. The primary method was IVW, while MR Egger assessed the presence of pleiotropy with the intercept term. A zero-intercept term indicates horizontal pleiotropy. Cochran’s Q tests were conducted to quantify IV heterogeneity and leave-one-out analyses were employed to identify potential IV heterogeneity.

For exposures displaying significant associations with outcomes, PhenoScanner was utilized to examine each SNP and determine whether the association could be attributed to pleiotropy. To establish a causal association between gut microbiota and infectious diseases, inverse MR analyses were conducted. We applied Bonferroni correction for multiple testing, setting the nominal significance threshold at *p* < 0.05. Corrected thresholds were defined as follows: genera: 0.05 / 131 (3.81E-04), families: 0.05 / 35 (1.4E-03), orders: 0.05 / 20 (2.5E-03), classes: 0.05 / 16 (3.1E-03), and phyla: 0.05 / 9 (5.56E-03).

All the analyses were conducted in R Studio environment (R version 4.3.1) by using TwoSampleMR package (version 0.5.7). PhenoScanner were available at http://www.phenoscanner.medschl.cam.ac.uk/.

### 2.5. *Post hoc* analysis

To further verify the association between gut microbiota and intestinal infections, we performed MR analysis on statistics from UK Biobank. To access the possibility of reverse cause, bi-directional MR analyses were also performed.

## 3. Results

### 3.1. Main analysis

After multiple testing correction, there are several statistically significant associations between gut microbiota and infectious diseases (Additional File 1. Supplementary Table S2, Additional File 2. Supplementary Table S2, Additional File 3. Supplementary Table S2).

**Figure 2:**
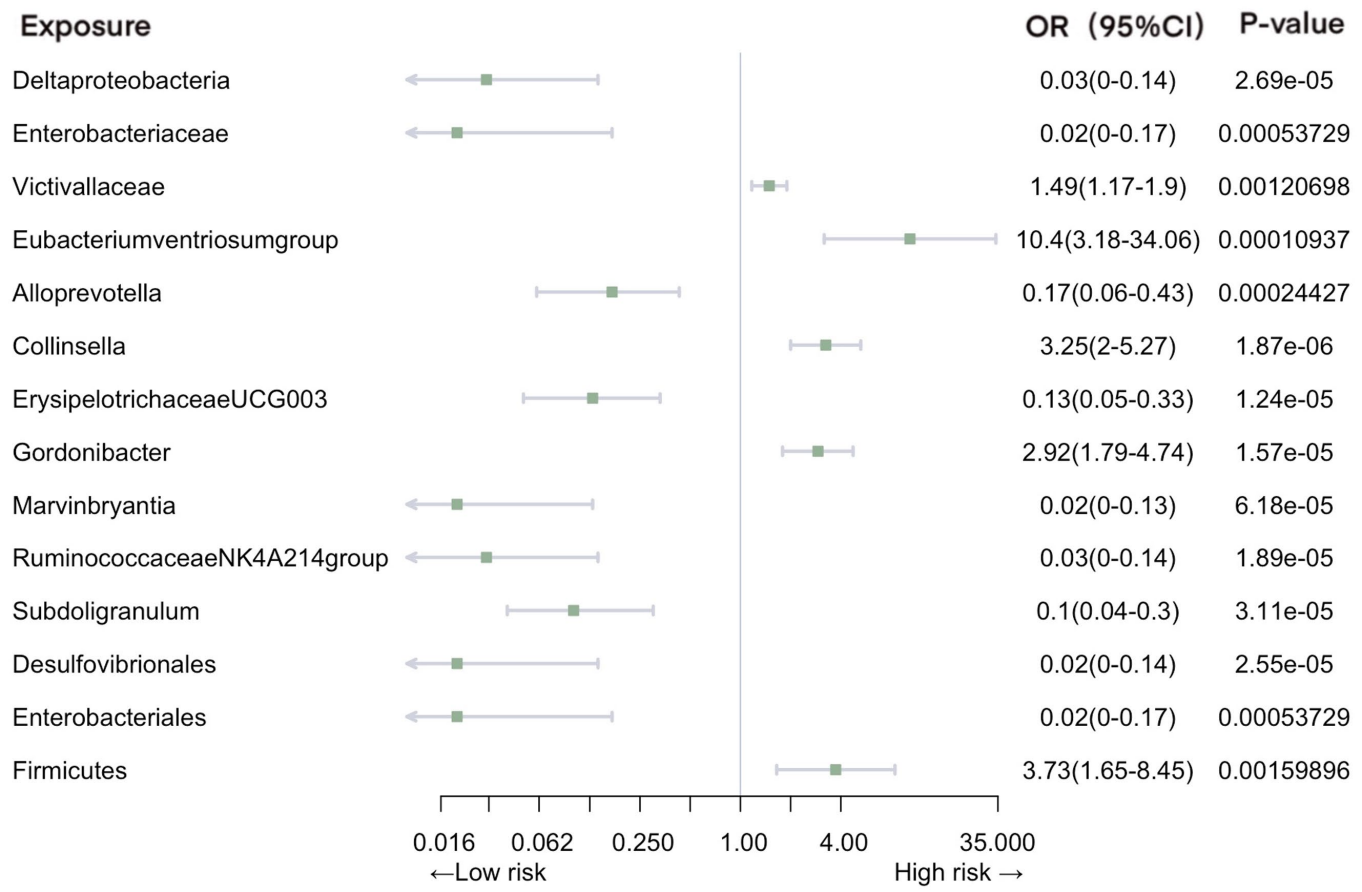
Mendelian randomization analyses of gut microbiota on the risk of intestinal infection. Legend: Forest plot of IVW analyses.

Our analysis revealed a total of 14 gut microbiotas associations with intestinal infection, encompassing 1 class, 2 families, 8 genera, 2 orders, and 1 phylum. Employing IVW evaluation, we identified significant associations (Figure 2). Notably, class *Deltaproteobacteria* (OR = 0.026, 95% CI: 0.0046–0.14, *p* = 2.69E-05), family *Enterobacteriaceae* (OR = 0.018, 95% CI: 0.0018–0.17, *p* = 5.37E-04), genus *Alloprevotella* (OR = 0.17, 95% CI: 0.063–0.43, *p* = 1.87E-06), genus *ErysipelotrichaceaeUCG003* (OR = 0.13, 95% CI: 0.054–0.33, *p* = 1.24E-05), genus *Marvinbryantia* (OR = 0.019, 95% CI: 0.027–0.13, *p* = 6.18E-05), genus *Ruminococcaceae NK4A214 group* (OR = 0.025, 95% CI: 0.004–0.14, *p* = 1.89E-06), genus *Subdoligranulum* (OR = 0.10, 95% CI: 0.036–0.30, *p* = 3.11E-05), order *Desulfovibrionales* (OR = 0.024, 95% CI: 0.0042–0.14, *p* = 2.55E-05), and order *Enterobacteriales* (OR = 0.024, 95% CI: 0.0042–0.14, p = 2.55E-05) exhibited protective effects against intestinal infection. Conversely, family *Victivallaceae* (OR = 1.49, 95% CI: 1.17–1.90, *p* = 1.21E-03), genus *Eubacteriumventriosumgroup* (OR = 10.40, 95% CI: 3.18–34.06, *p* = 1.09E-04), genus *Gordonibacter* (OR = 2.92, 95% CI: 1.79–4.74, *p* = 1.57E-05), genus *Collinsella* (OR = 3.25, 95% CI: 2.00–5.27, *p* = 1.87E-06), and phylum *Firmicutes* (OR = 3.73, 95% CI: 1.65–8.45, *p* = 1.60E-03) were associated with an increased risk of intestinal infection.

**Figure 3:**
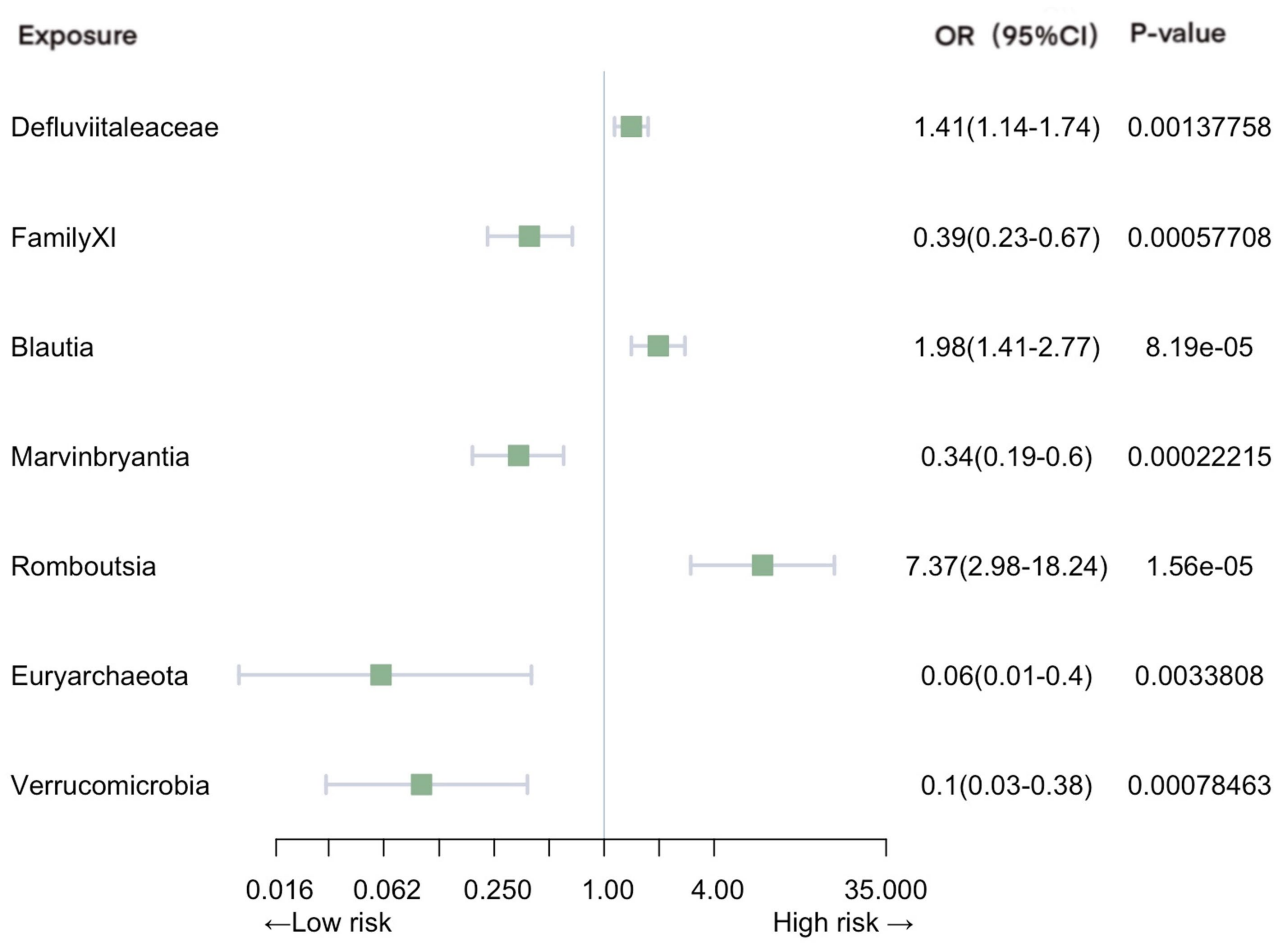
Mendelian randomization analyses of gut microbiota on the risk of pneumonia. Legend: Forest plot of IVW analyses.

Significant associations with pneumonia were observed in gut microbiota from 2 families, 4 genera, and 2 phyla (Figure 3). Our analysis revealed positive causal effects, from phylum *Euryarchaeota* (OR = 0.063, 95% CI: 0.010–0.40, *p* = 3.38E-03), phylum Verrucomicrobia (OR = 0.099, 95% CI: 0.026–0.38, *p* = 7.85E-04), family *FamilyXI* (OR = 0.39, 95% CI: 0.23–0.67, *p* = 5.77E-04), and genus *Marvinbryantia* (OR = 0.34, 95% CI: 0.19–0.60, *p* = 2.22E-04). In contrast, family *Defluviitaleaceae* (OR = 1.41, 95% CI: 1.14–1.74, *p* = 1.38E-03), genus *Blautia* (OR = 1.98, 95% CI: 1.41–2.77, *p* = 8.19E-05), and genus *Romboutsia* (OR = 7.37, 95% CI: 2.98–18.23, *p* = 1.56E-05) were associated with negative effects.

**Figure 4:**
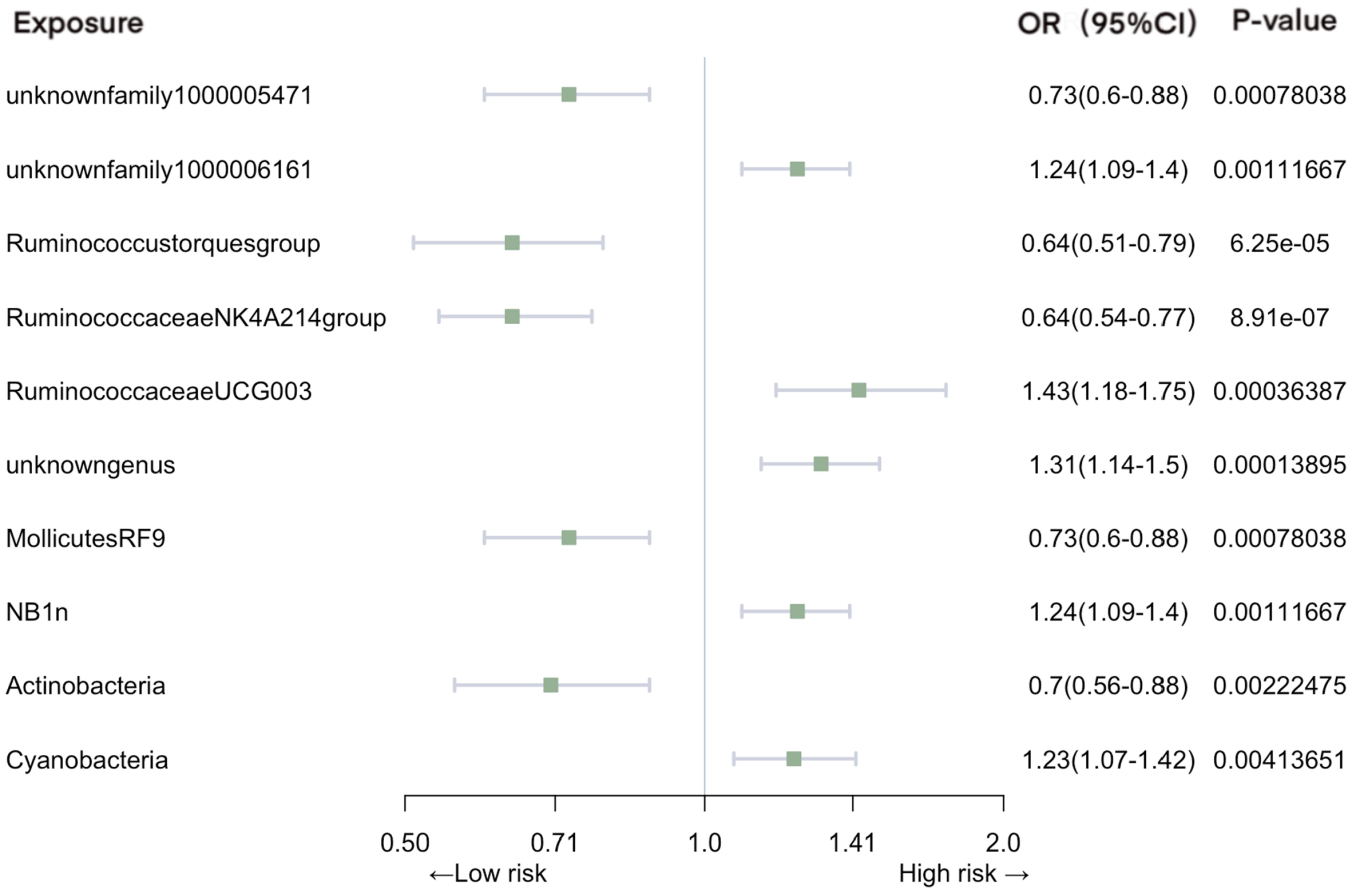
Mendelian randomization analyses of gut microbiota on the risk of urinary tract infection. Legend: Forest plot of IVW analyses.

In the context of urinary tract infections, our analysis identified several significant associations (Figure 4). Family *unknownfamily1000005471* (OR = 0.73, 95% CI: 0.60–0.88, *p* = 7.80E-04), genus *Ruminococcustorquesgroup* (OR = 0.64, 95% CI: 0.51–0.79, *p* = 6.25E-05), genus *Ruminococcaceae NK4A214 group* (OR = 0.64, 95% CI: 0.54–0.77, *p* = 8.91E-07), order *MollicutesRF9* (OR = 0.73, 95% CI: 0.60–0.88, p = 7.80E-04), and phylum *Actinobacteria* (OR = 0.70, 95% CI: 0.56–0.88, p = 2.22E-03) were associated with a reduced risk of urinary tract infections. In contrast, family *unknownfamily1000006161* (OR = 1.24, 95% CI: 1.09–1.40, *p* = 1.12E-03), genus *RuminococcaceaeUCG003* (OR = 1.43, 95% CI: 1.18–1.75, *p* = 3.64E-04), genus *unknowngenus* (OR = 1.31, 95% CI: 1.14–1.50, *p* = 1.39E-04), order *NB1n* (OR = 1.24, 95% CI: 1.09–1.40, p = 1.12E-03), and phylum *Cyanobacteria* (OR = 1.23, 95% CI: 1.07–1.42, *p* = 4.14E-03) exhibited an increased association with the risk of urinary tract infections.

### 3.2. Sensitivity analysis

The MR-Egger, weighted median, simple mode, and weighted mode methods were selectively applied exclusively to gut microbiota instances where the number of SNPs were more than 3. This approach was informed by *F*-statistic power exceeding 10, effectively eliminating weak instrumental bias (Additional File 1. Supplementary Table S1, Additional File 2. Supplementary Table S1, Additional File 3. Supplementary Table S1). All analyses consistently yielded congruent beta directions, substantially reinforcing the robustness of our primary analyses (Additional File 1. Supplementary Table S3, Additional File 2. Supplementary Table S3, Additional File 3. Supplementary Table S3). Conspicuously, no instances of heterogeneity or pleiotropy emerged in our main analyses across the three infectious diseases (Additional File1. Supplementary Table S5, Additional File 2. Supplementary Tables S5, S6, Additional File 3. Supplementary Tables S5, S6). Upon employing PhenoScanner, we identified associations between rs11597285 and rs12251396 and eosinophil percentage in blood. Remarkably, leave-one-out analyses indicated the absence of any individual SNP significantly associated with all three infectious diseases, thereby signifying a dearth of pleiotropic effects (Additional File 1. Supplementary Table S4, Additional File 2. Supplementary Table S4, Additional File 3. Supplementary Table S4).

### 3.3. *Post hoc* analysis

Our comprehensive bi-directional MR analyses ruled out any reversed causal relationships between infectious diseases and gut microbiota. To uncover shared effects within specific gut microbiota, we leveraged the robust GWAS statistics from the UK Biobank. Remarkably, our findings highlighted nominal associations: order *NB1n* exhibited a connection with urinary tract infection, while genus *ErysipelotrichaceaeUCG003* displayed a distinctive tie to intestinal infection.

Additionally, our investigation delved into the implications of two distinct SNPs associated with eosinophil counts. The MR analysis covering eosinophilic diseases and intestinal infections yielded results that did not support the presence of any causal association (*p* = 0.908).

## 4. Discussion

In this TwoSample MR analysis, we integrated 211 diverse microbiota types from the extensive MiBioGen consortium’s meta-analysis GWAS, along with summary statistics from FinnGen R9 release data for three infectious diseases, aiming to assess their causal relationship. Our findings unveiled a range of protective gut microbiotas alongside a few associated with increased risk. Specific microbial communities proficient in butyrate production, including the families *Ruminococcaceae* and *Lachnospiraceae*, manifested a duality of advantageous and adverse effects. The impact of particularly microbial families, including *Erysipelotrichaceae* and *Defluviitaleaceae*, was also identified. These results could potentially introduce a novel approach for addressing infectious diseases in human populations.

An increasing number of observational studies has highlighted the link between gut microbiota and infections (Schuijt et al., 2016; Ducarmon et al., 2019; Wang et al., 2019). The gut microbiota plays a pivotal role in bolstering resistance against bacterial infections (Ducarmon et al., 2019), while it can also contribute to the disruption of the gut barrier, potentially leading to infections originating from the gut (Wang et al., 2019). For instance, *Erysipelotrichaceae* has shown a strong correlation with host lipid metabolism, thus potentially influencing inflammation (Kaakoush, 2015).

A prior investigation revealed that *Desulfocibrio desulfuricans* could contribute to aneurysm infections (Fujihara et al., 2023). Additionally, another study noted that the order *Desulfovibrionales* and the family *Desulfovibrionaceae* were linked to an increased risk of chronic obstructive pulmonary disease (Wei et al., 2023). In the study we conducted, the results indicate that the order *Desulfovibrionales* and the class *Deltaproteobacteria* served as safeguards in preventing intestinal infections. One potential explanation is their influence on RNA transcription (Wang W. et al., 2022), subsequently impacting gut protein levels (Langwig et al., 2022; Hu et al., 2023a). However, the exact mechanisms through which these microorganisms influence the development of gut infections require more in-depth investigation. Further research is needed to better understand how these specific bacterial groups contribute to safeguarding the gut against infections and to uncover the underlying molecular and biological processes involved.

*Prevotella*, recognized as the second most abundant family in the human oral cavity, frequently maintains a high prevalence within gut microbiota (Tett et al., 2021). Some observations suggest that specific *Prevotella* strains might function as clinical pathobionts, contributing to human diseases by elevating inflammation risk and promoting Th1 immune responses (Larsen, 2017). Conversely, a distinct study found a decrease in *Prevotella* abundance in pediatric Crohn’s disease (Lewis et al., 2015). Parallelly, our study highlights that the genus *Alloprevotella* within the *Prevotella* family exhibits a protective effect. Genus *ErysipelotrichaceaeUCG003* as another defensive factor, aligns with an observation that noted lower family *Erysipelotrichaceae* abundance in patients experiencing Crohn’s recurrence (Dey et al., 2013). Furthermore, the family *Enterobacteriaceae* within the order *Enterobacteriales*, known for antibiotic resistance (Nordmann et al., 2012), was found to confer a protective effect. This contrasts with previous research that implicated it in acute knee prosthetic joint infections (Martínez-Pastor et al., 2010).

Members of *Lachnospiraceae* exhibit anaerobic, fermentative, and chemoorganotrophic characteristics, primarily generating butyrate—an ending product of bacterial metabolism known to regulate gut inflammatory processes. Despite the repeated identification of this family’s taxa for their ability to produce beneficial metabolites for humans, their abundance paradoxically increases in the intestinal lumen of a subset of patients (Vacca et al., 2020). Within this context, our study demonstrates noteworthy correlations: genus *Marvinbryantia* exhibited a positive effect on both intestinal infections and pneumonia, while genus *Blautia*—previously reported to associated with beneficial anti-inflammatory effects—displayed a negative influence pneumonia (Vacca et al., 2020; Liu et al., 2021).

A study previously discovered a higher abundance of the *Victivallaceae* family in patients who developed AIDS (Chen et al., 2021). Correspondingly, our findings paralleled this observation, with *Victivallaceae* emerging as a risk factor for intestinal infections. Similarly, the *Firmicutes* phylum also stood out as a risk factor. Despite an earlier study indicating a positive influence of the *Eubacterium ventriosum group* genus on Inflammatory Bowel Disease (Liu et al., 2022), our research contradicts this by identifying it as a risk factor for intestinal infections. The intricate mechanism through which the *Eubacterium ventriosum group* affects infections necessitates further investigation. Moreover, the genus *Gordonibacter*, associated with inflammatory responses in both birds and mammals (Nair et al., 2021), was isolated from a Crohn’s disease patient (Wurdemann et al., 2009) and emerged as a risk factor for intestinal infections in our study. The presence of the genus *Collinsella* also exerted a negative influence.

The phyla *Euryarchaeota* and *Verrucomicrobia* were identified as protective elements against pneumonia. Nonetheless, due to their extensive diversity and limited prior observations, substantial research is warranted to unveil their underlying mechanisms and specific species involved. We identified the Family *Defluviitaleaceae* as a potential risk factor for pneumonia within our study. Significantly, a recent observation linked the genus *Romboutsia* to bone marrow necrosis induction, and our research further identifies it as a risk factor for pneumonia (Seviar et al., 2022).

The order *MollicutesRF9* and class *Actinobacteria* served as factors contributing to urinary tract infection protection. *Cyanobacteria*, known for their significant impact on water quality and ecosystems, have been previously suggested to produce highly neurotoxic cyanotoxins that exert their effects through diverse molecular mechanisms. In our study, we identified *Cyanobacteria* as a risk factor for urinary tract infections (Sini et al., 2021). Additionally, the lack of order *NB1n* was also associated with an elevated risk.

Throughout our comprehensive study, we observed a distinct group of gut microbiotas that exhibited a strong correlation with both intestinal and urinary tract infections—namely, the *Ruminococcaceae* family. As a typical butyrate-producing bacteria in mammals, its influence extends to bacterial metabolism, and a decrease in its abundance has been linked to infection susceptibility (He et al., 2022). *RuminococcaceaeUCG003*, a key genus, was implicated in chronic insomnia and cardiometabolic diseases, while the *Ruminococcustorques group* has been found to be markedly prevalent in chronic infections (Jiang et al., 2022; Tran et al., 2023). Additionally, the *Ruminococcaceae NK4A214 group* has been associated with diarrhea (Gu et al., 2022). Intriguingly, our findings indicated a protective role for the *Ruminococcaceae NK4A214 group* against both intestinal and urinary tract infections, while *Subdoligranulum* exhibited a negative impact on intestinal infection. The *Ruminococcustorques group* demonstrated a protective effect against urinary tract infections, whereas *Ruminococcaceae UCG003* increased the risk. We postulate that this family plays a crucial role in mediating the overall gastrointestinal environment through its butyrate production. Nonetheless, the innate mechanism behind this phenomenon remains contentious, necessitating further trials to unravel its complexities.

This significant revelation of two previously unidentified SNPs in our analysis adds a new layer of complexity to our understanding. These unique genetic variations may hold the key to unlocking insights into the gene’s involvement in the innate pathway, shedding light on its potential role in infectious disease susceptibility (Hu et al., 2023b). As we embark on future investigations, delving deeper into the intricate molecular mechanisms behind these associations promises to unveil novel therapeutic targets and advance our knowledge of genetic contributions to disease risk (Wang et al., 2023).

Our study boasts several strengths and limitations. Employing MR analysis, we mitigated confounding risks commonly encountered in observational trials. Furthermore, our study encompassed a larger sample size compared to conventional RCTs. However, certain limitations are present. The GWAS data used for analysis predominantly originated from individuals of European ancestry, potentially limiting the generalizability of our results to other racial groups. Additionally, our microbiota data from MibioGen was categorized into 211 groups, ranging from phylum to genus level, which precluded the identification of specific gut microbiota species. While the causal relationships established in FinnGen data did not replicate in the UK Biobank data, our *post hoc* analysis revealed just two overlapping microbiotas. We posit that regional differences between the UK and Finland, coupled with distinct phenotype definitions across the two cohorts, might have contributed to this discrepancy. Furthermore, due to our utilization of summary-level data, we were unable to ascertain potential overlap in participants between the exposure and outcome data, introducing the possibility of bias.

## 5. Conclusion

In this comprehensive exploration of the connection between gut microbiota and infectious diseases, our research has unveiled a diverse landscape of microbial influences on infection susceptibility. Utilizing the power of TwoSample Mendelian randomization, we identified several microbiota types that play pivotal roles in either enhancing or mitigating the risk of infections. Of particular interest is the *Ruminococcaceae* family, whose subgroups demonstrated both protective and risk-related impacts on infections. These findings underscore the intricate nature of microbiota-infection interactions and hold the promise of informing novel strategies for disease prevention and management. Moving forward, continued investigations are imperative to unravel the underlying mechanisms and clinical implications of these complex relationships.

## Data availability statement

The original contributions presented in the study are included in the article/Supplementary material, further inquiries can be directed to the corresponding author.

## Ethics statement

Written informed consent was obtained from the individual(s) for the publication of any potentially identifiable images or data included in this article.

## Author contributions

BL: Conceptualization, Formal analysis, Methodology, Software, Validation, Visualization, Writing – original draft. JM: Data curation, Validation, Writing – review & editing, Formal analysis. YB: Resources, Validation, Writing – original draft. ZF: Conceptualization, Investigation, Methodology, Project administration, Supervision, Writing – review & editing.
