# Supplementary material for: Casual effects of gut microbiota on risk of infections: a two-sample Mendelian randomization study
Source: Front Microbiol. 2023 Oct 10;14:1284723. doi: 10.3389/fmicb.2023.1284723 (PMC10595145; doi:10.3389/fmicb.2023.1284723)
Supplement: Supplementary file 4 [file Data_Sheet_1.PDF]

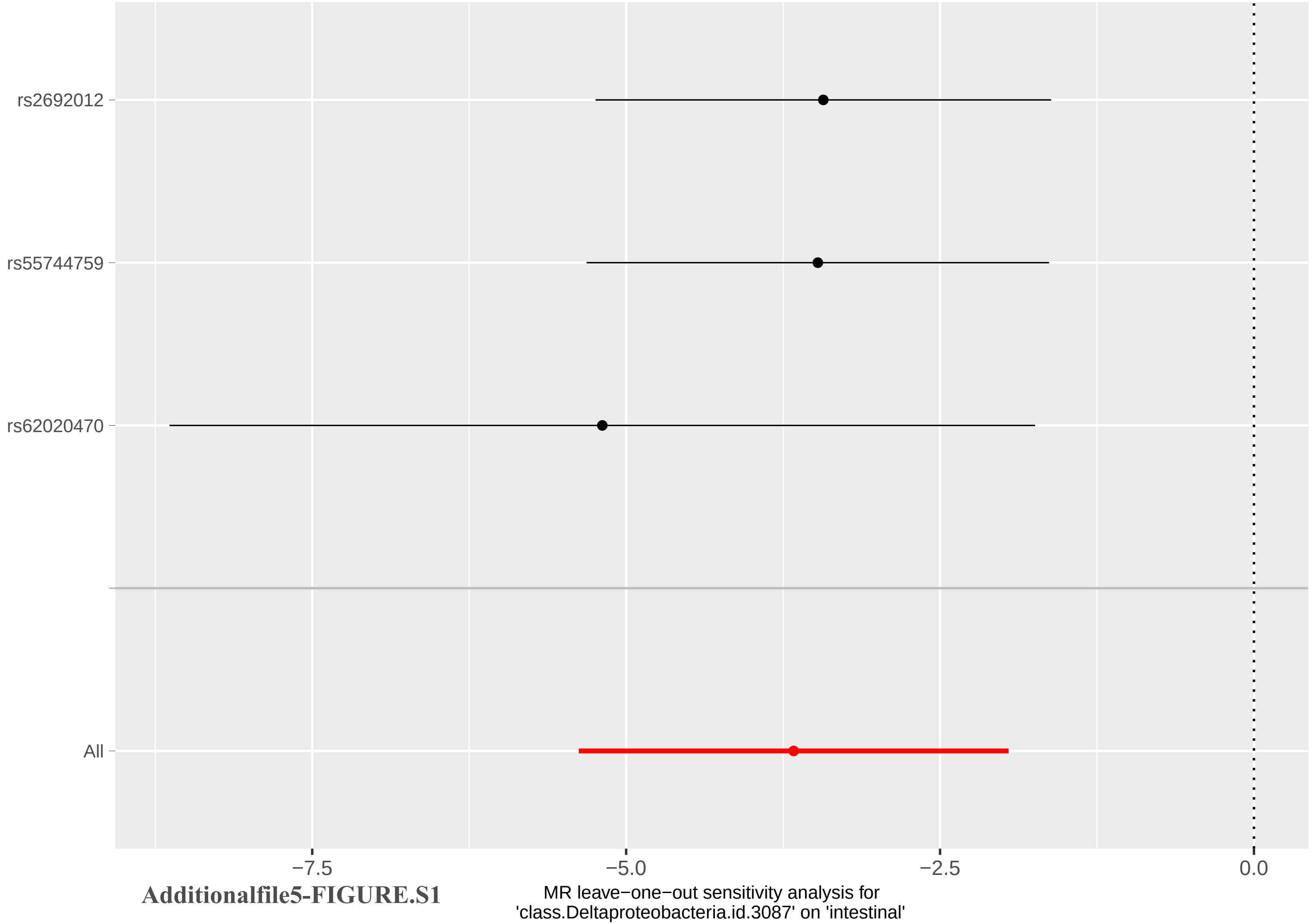

Additionalfile5-FIGURE.S1

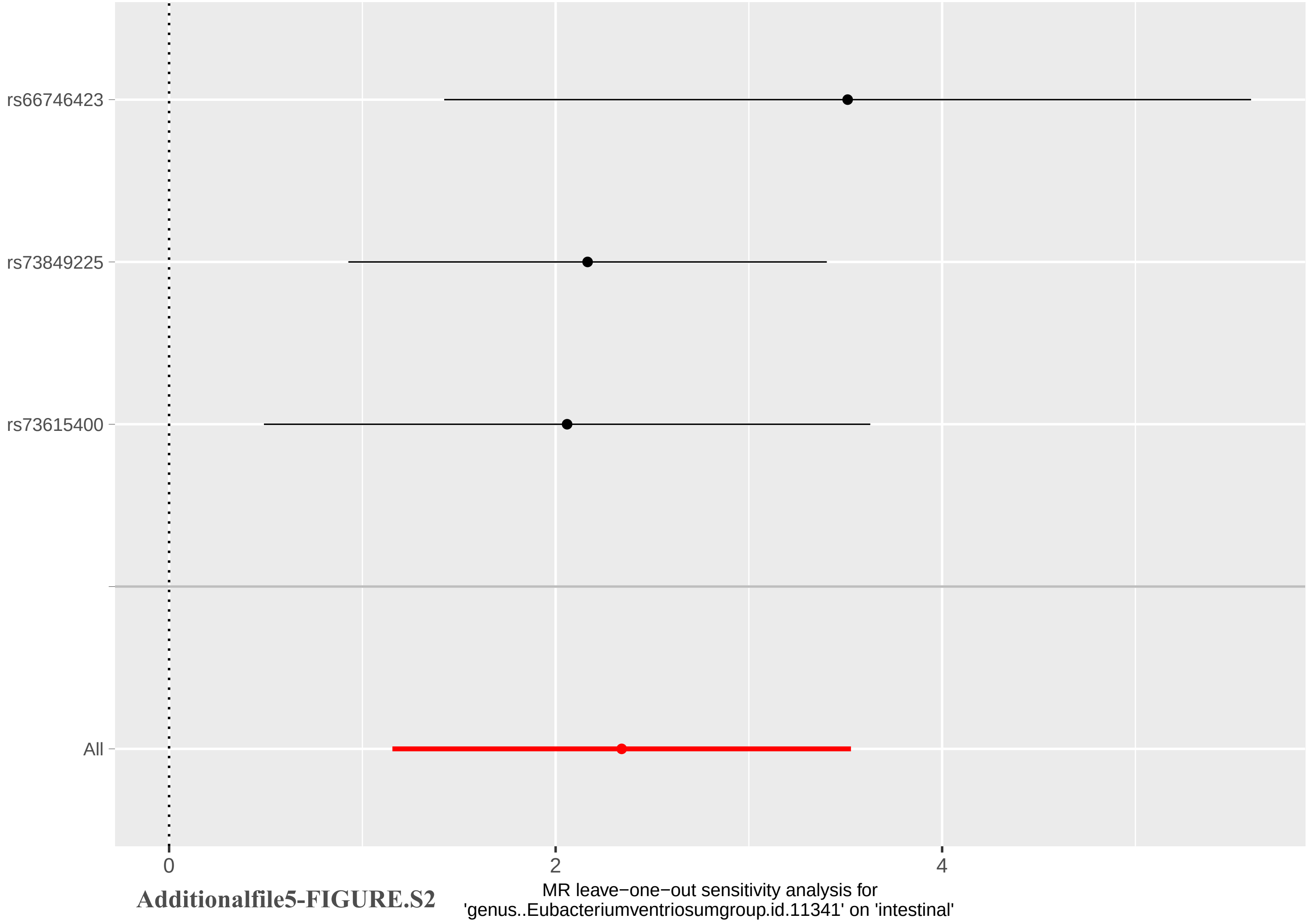

Additionalfile5-FIGURE.S2

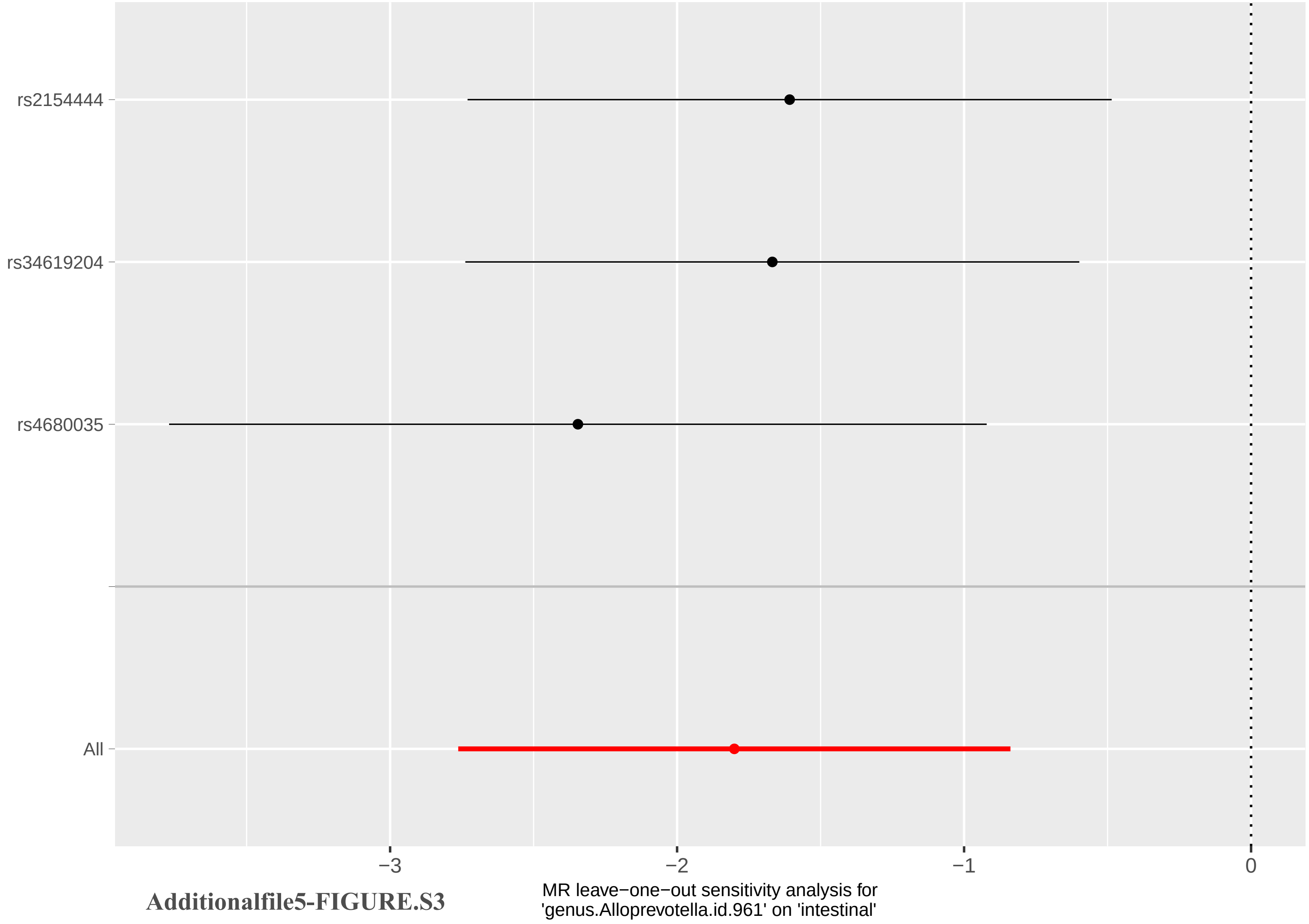

Additionalfile5-FIGURE.S3

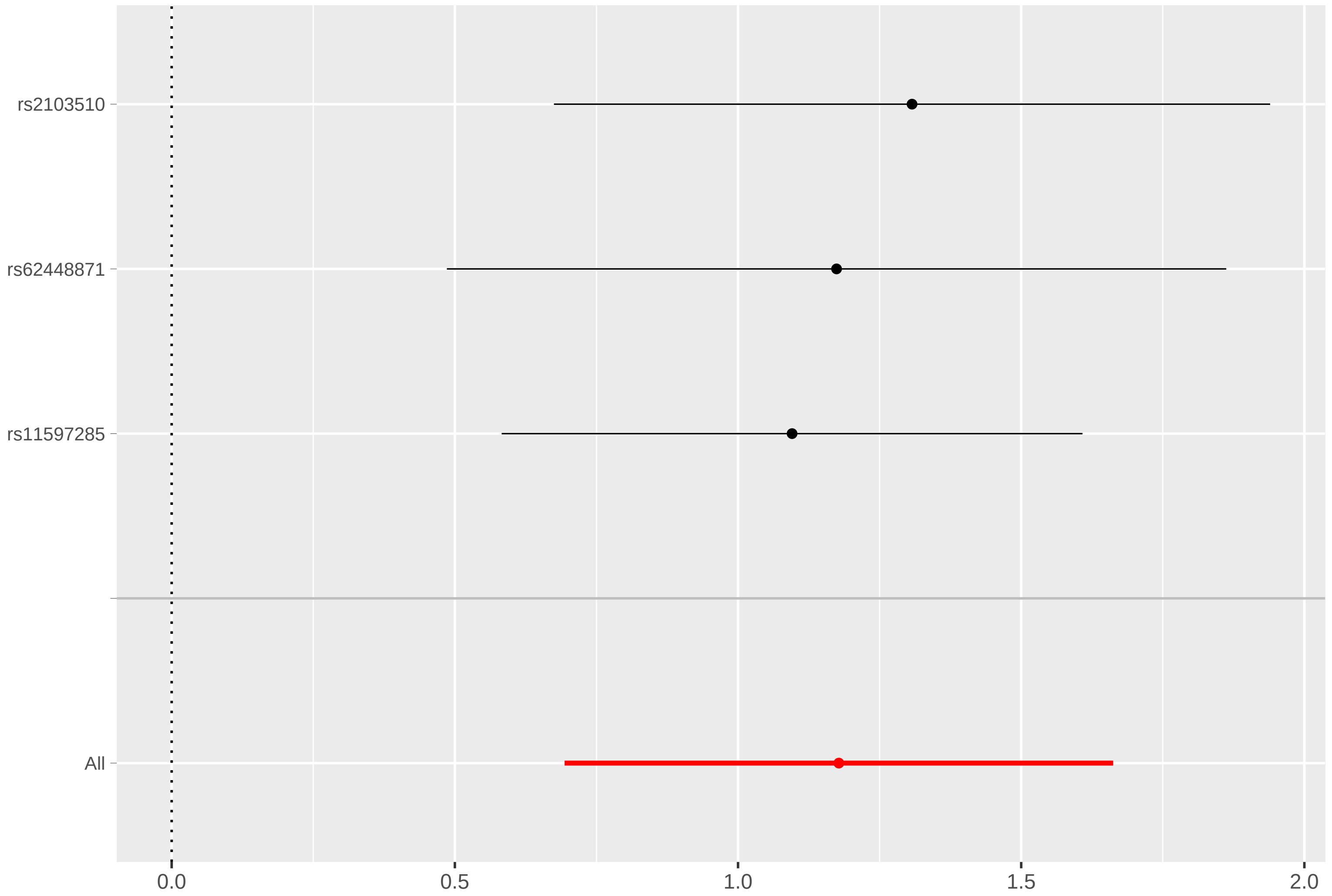

Additionalfile5-FIGURE.S4

MR leave−one−out sensitivity analysis for
'genus.Collinsella.id.815' on 'intestinal'

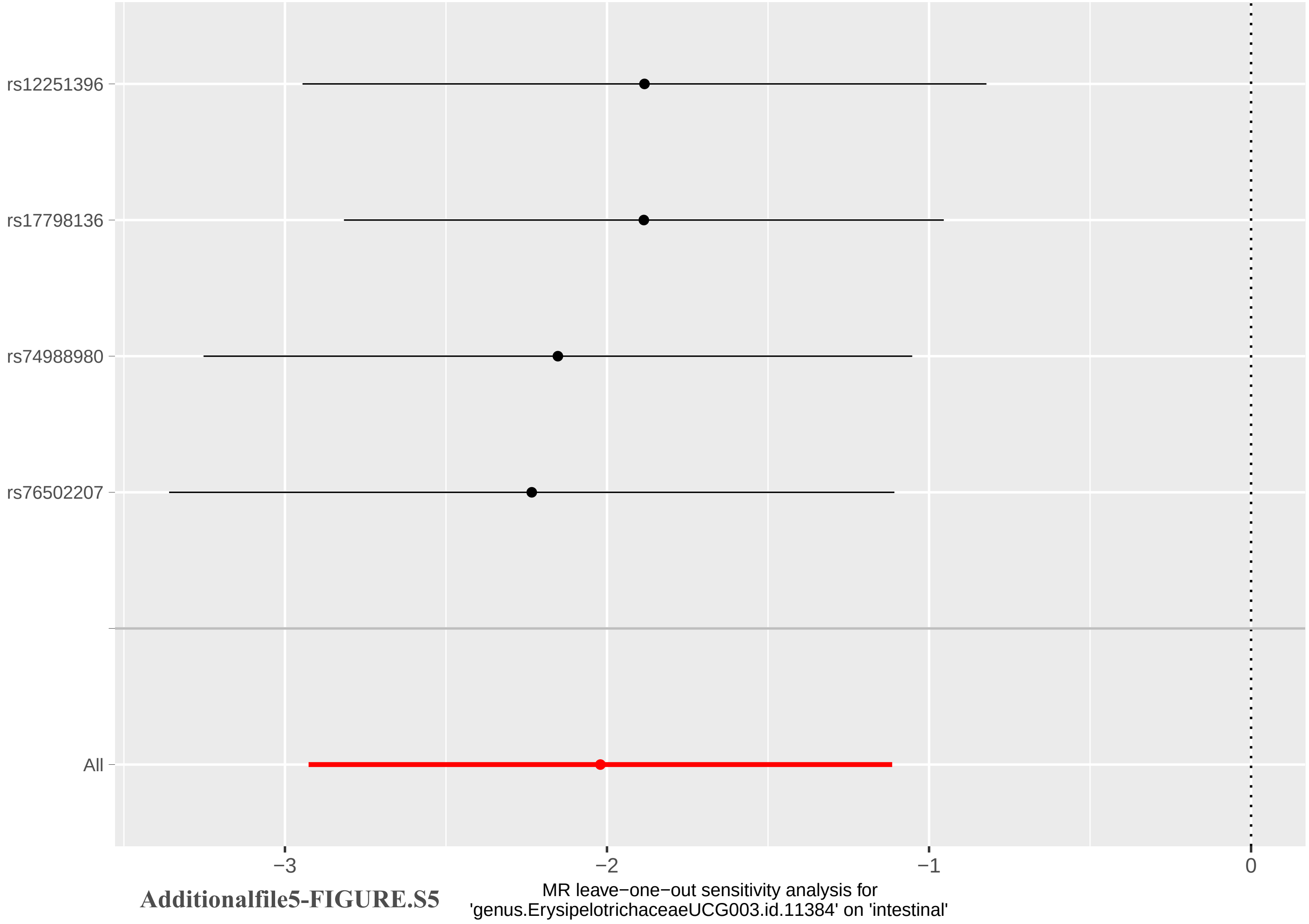

Additionalfile5-FIGURE.S5

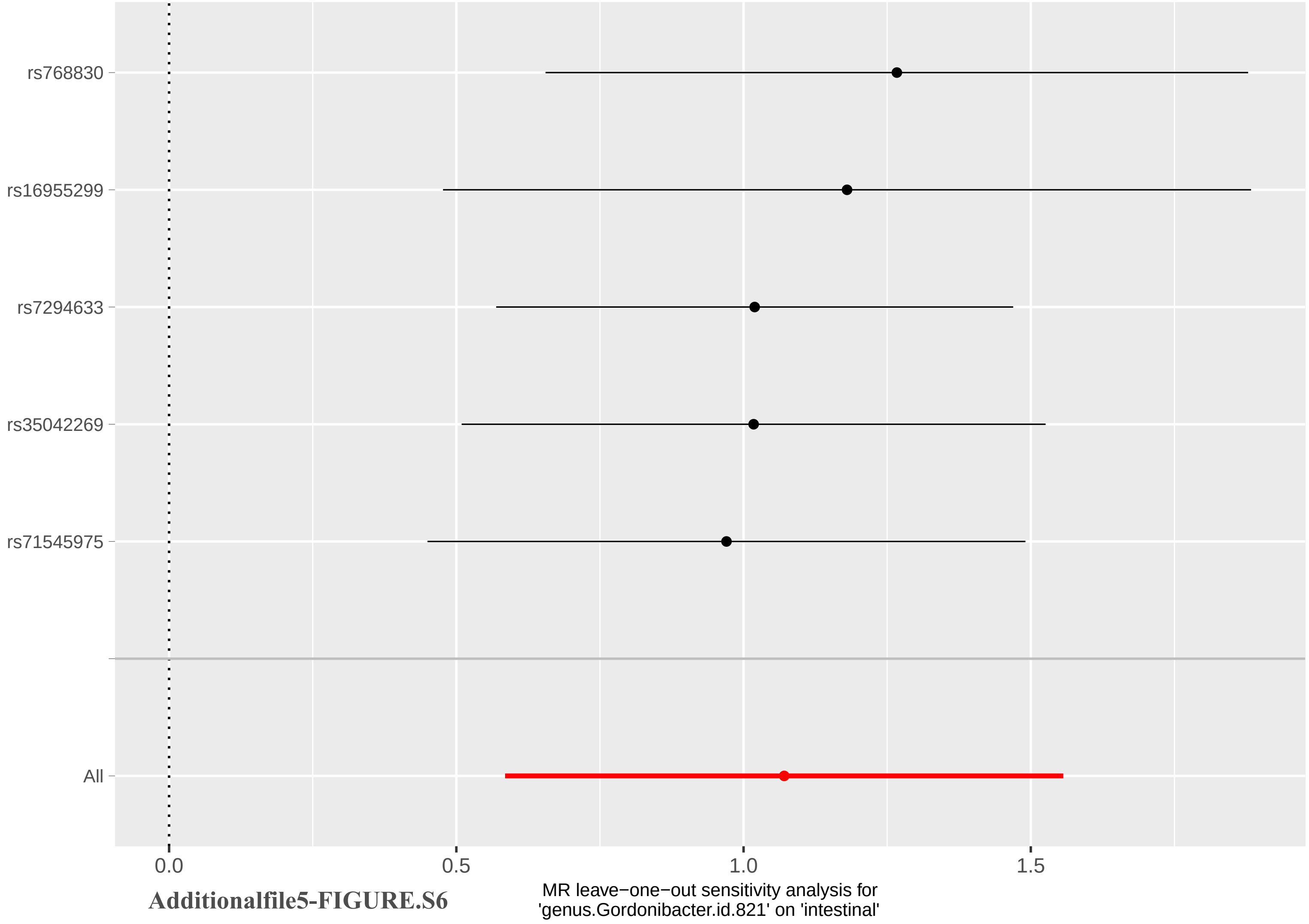

Additionalfile5-FIGURE.S6

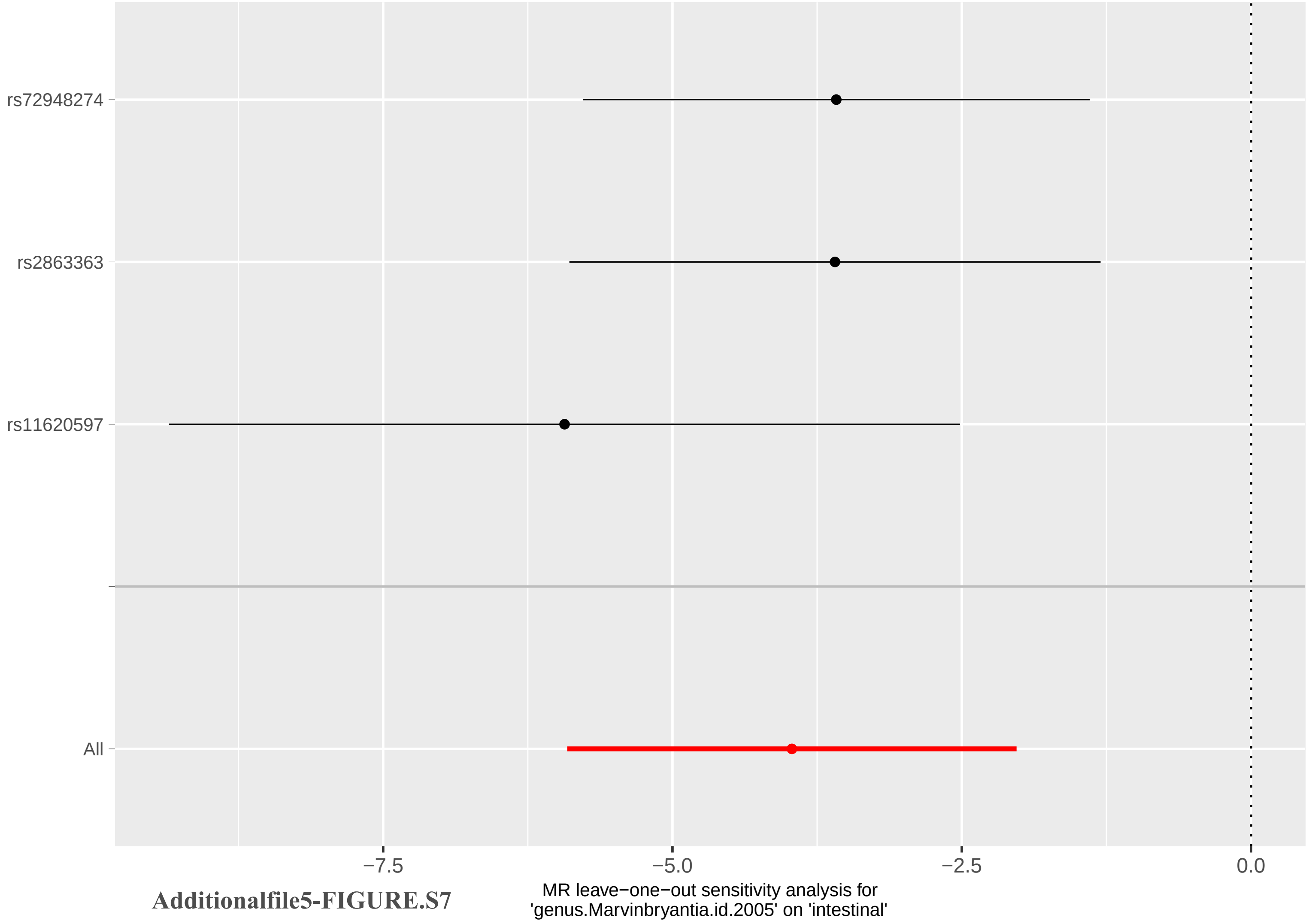

Additionalfile5-FIGURE.S7

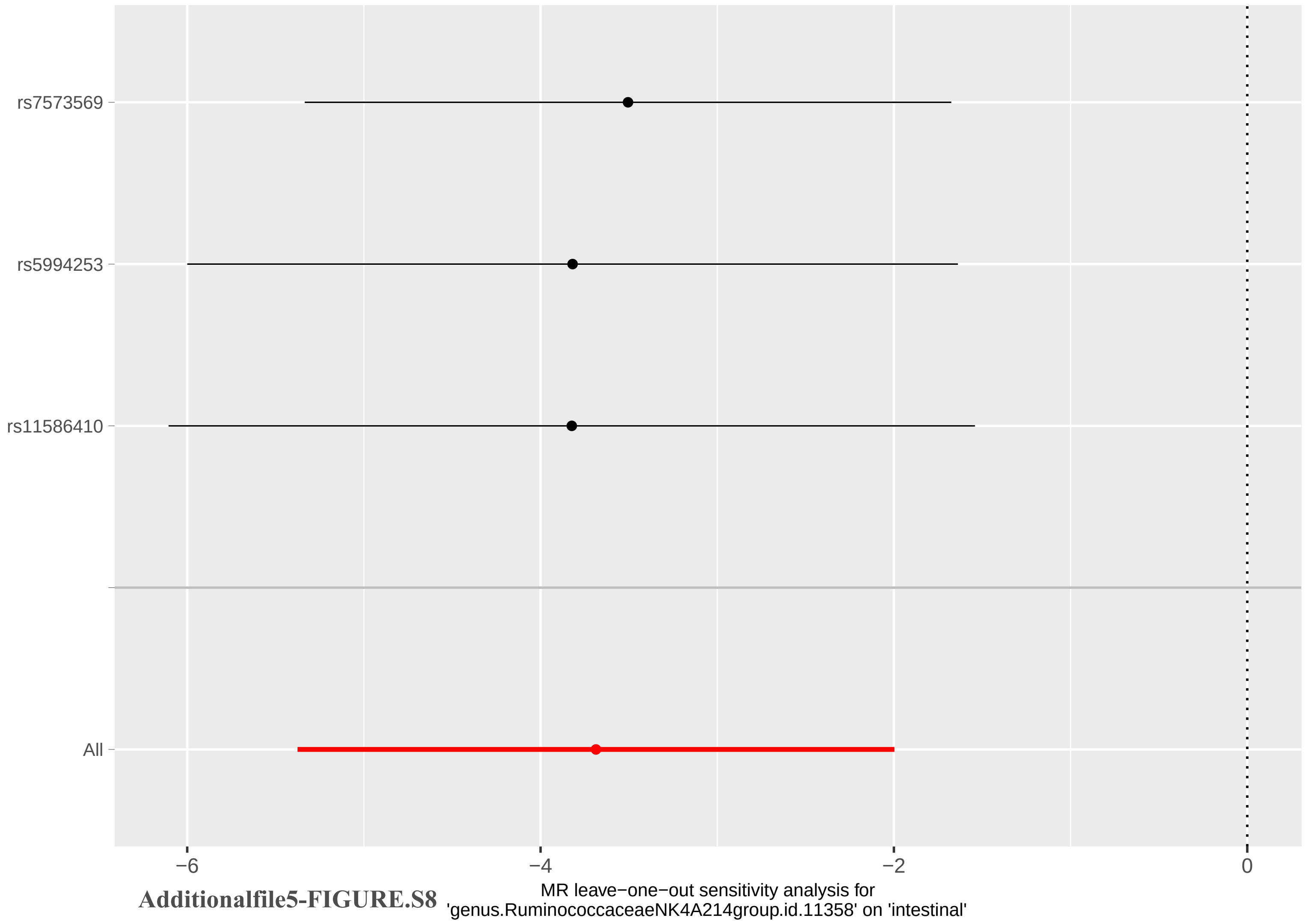

Additionalfile5-FIGURE.S8

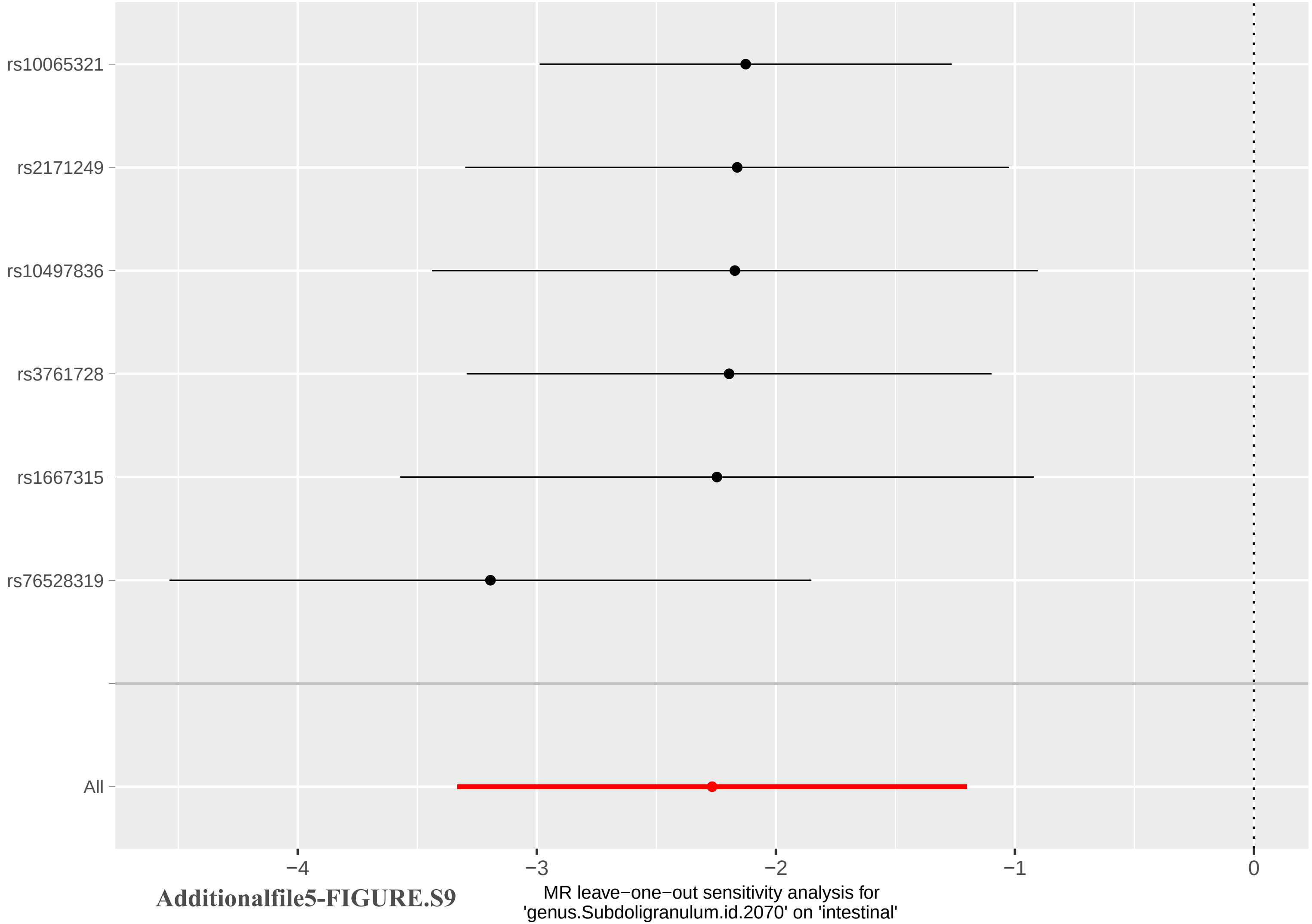

Additionalfile5-FIGURE.S9

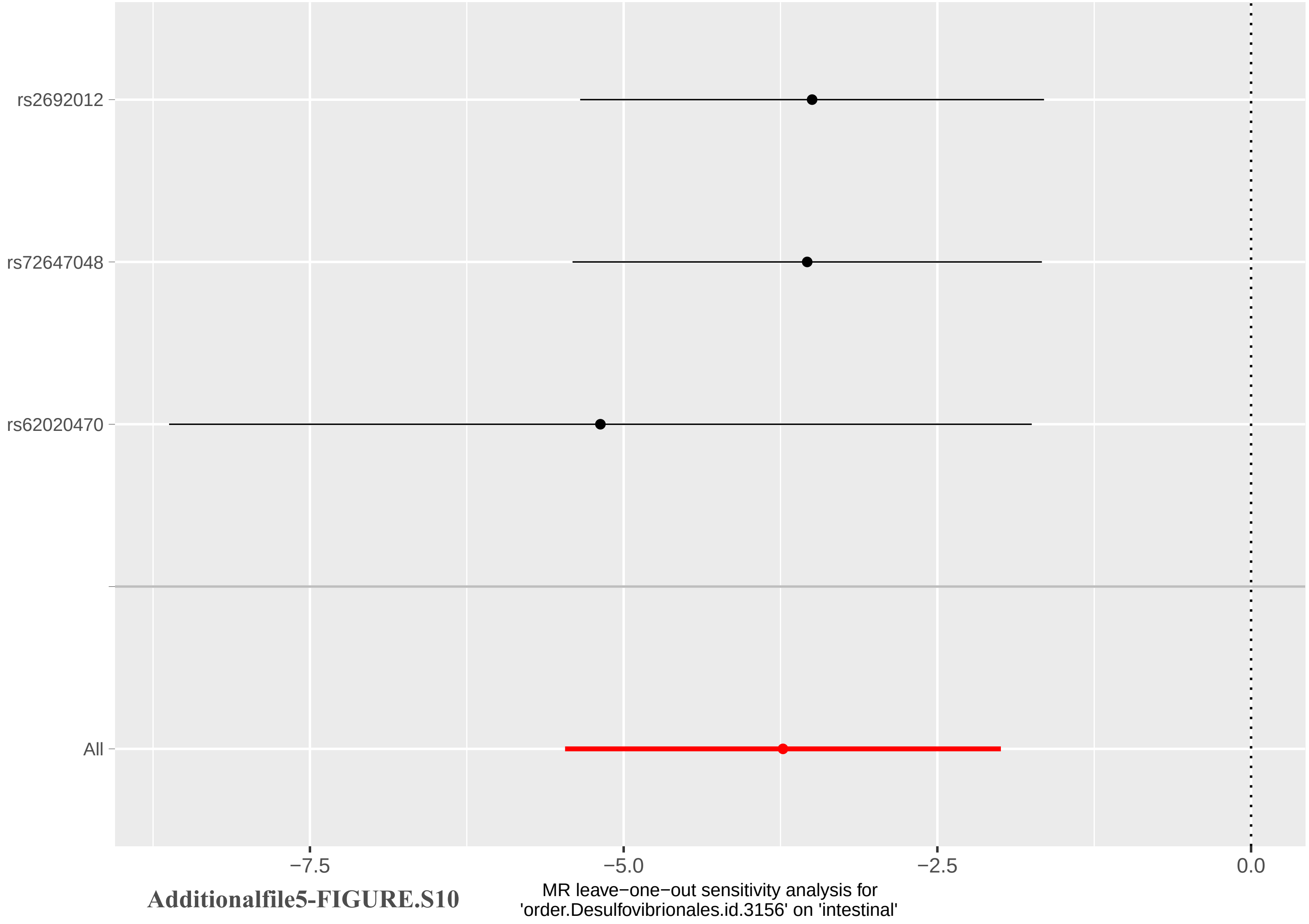

Additionalfile5-FIGURE.S10

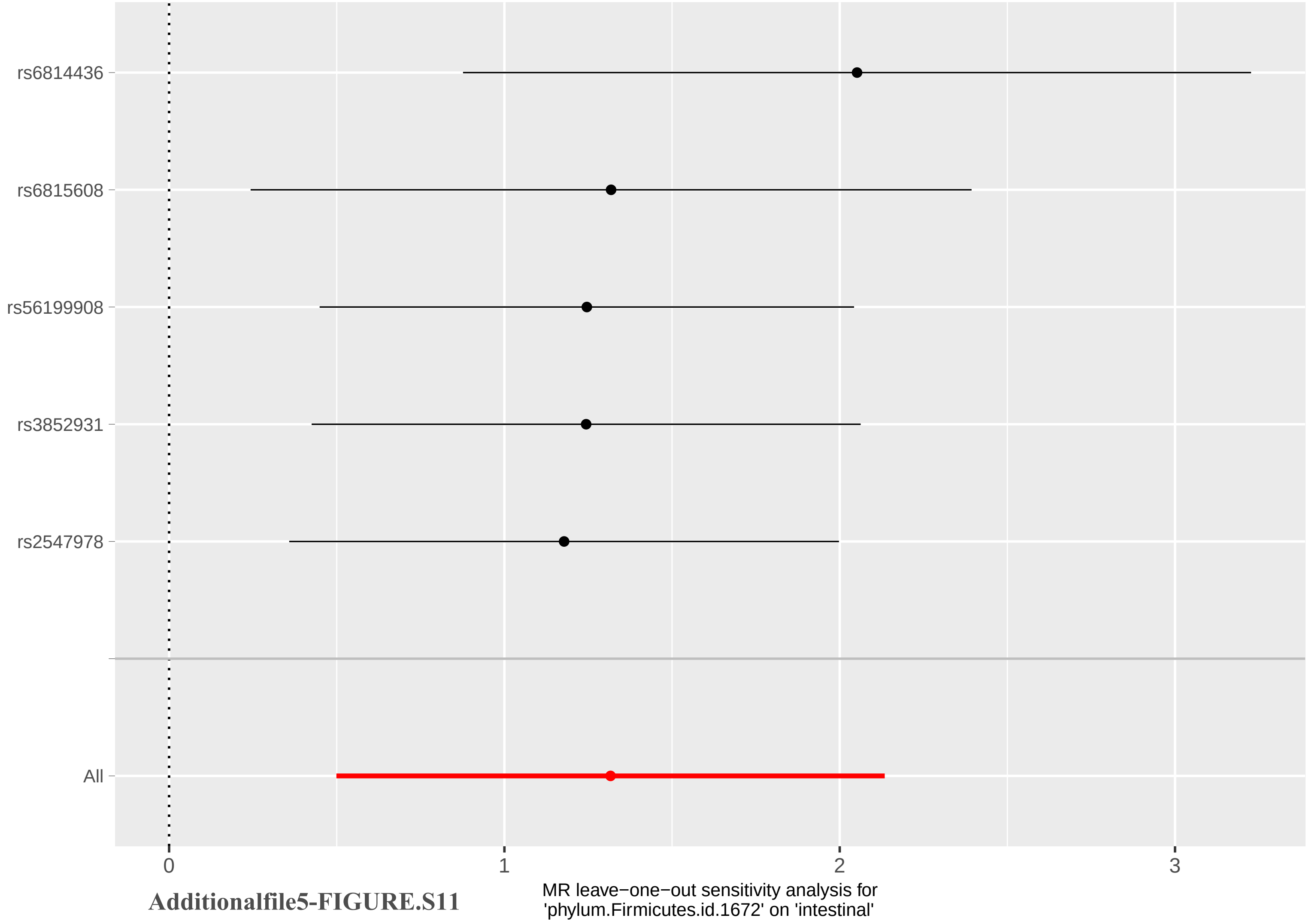

Additionalfile5-FIGURE.S11
