# Supplementary material for: Casual effects of gut microbiota on risk of infections: a two-sample Mendelian randomization study
Source: Front Microbiol. 2023 Oct 10;14:1284723. doi: 10.3389/fmicb.2023.1284723 (PMC10595145; doi:10.3389/fmicb.2023.1284723)
Supplement: Supplementary file 5 [file Data_Sheet_2.PDF]

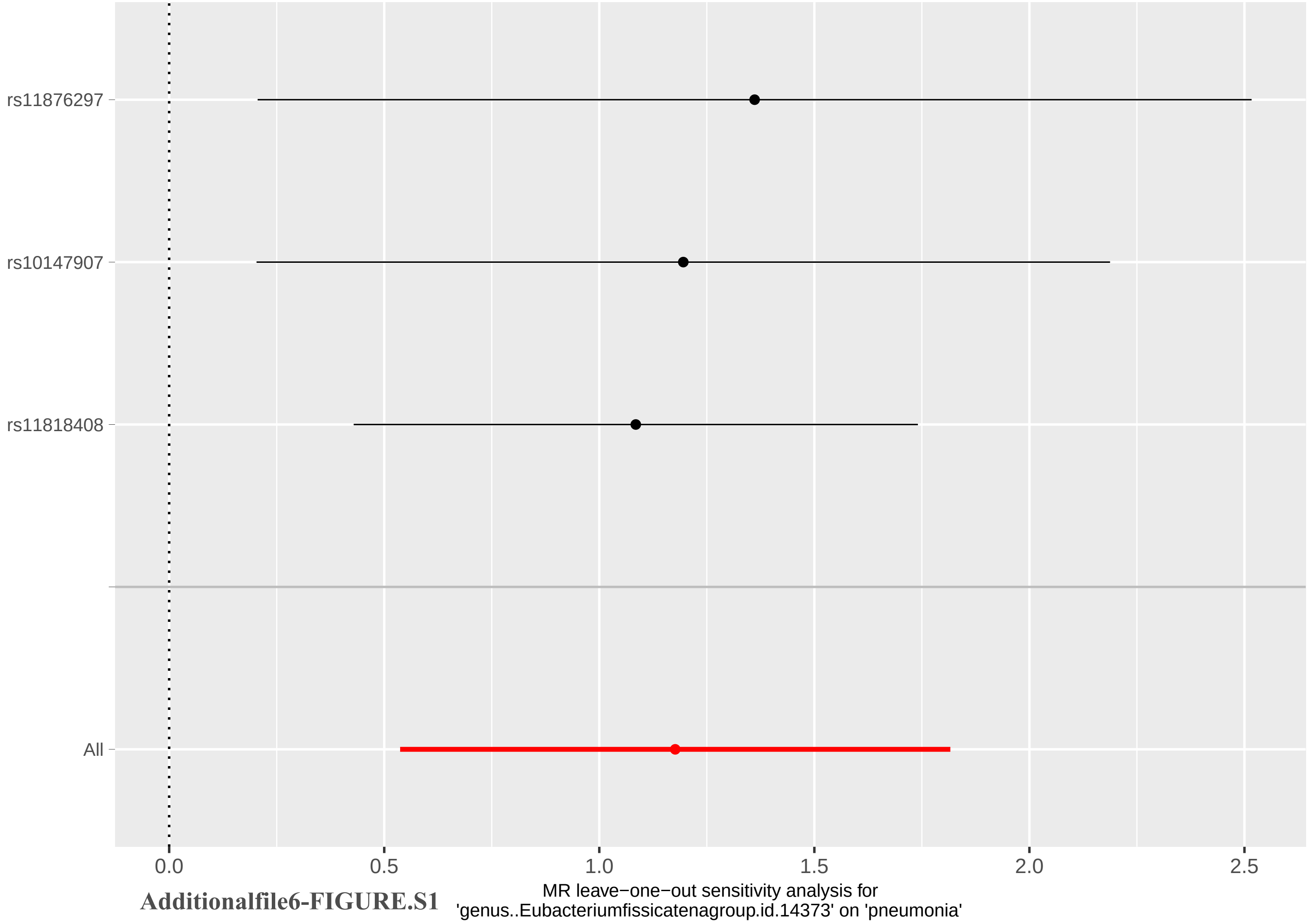

Additionalfile6-FIGURE.S1

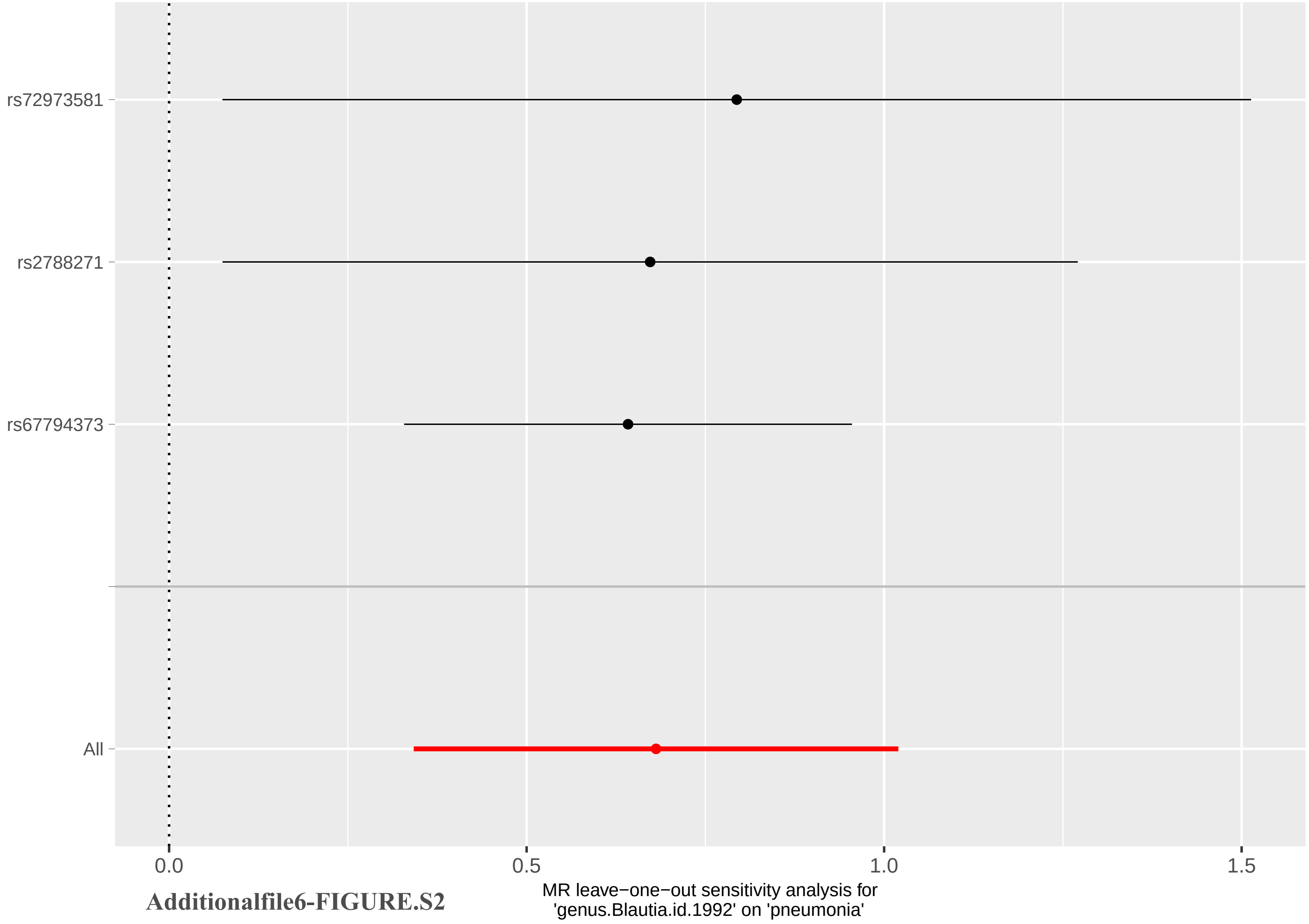

Additionalfile6-FIGURE.S2

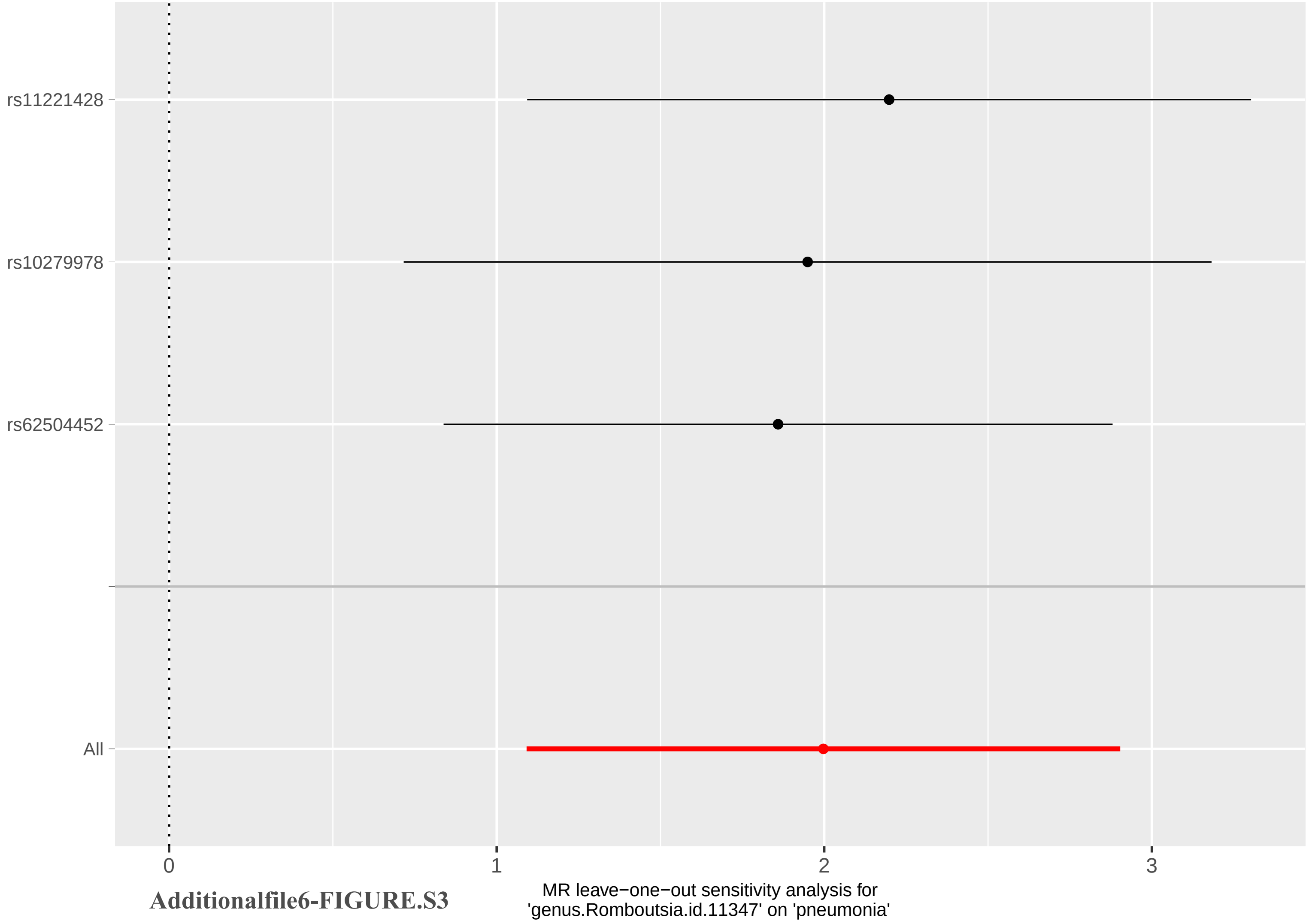

Additionalfile6-FIGURE.S3

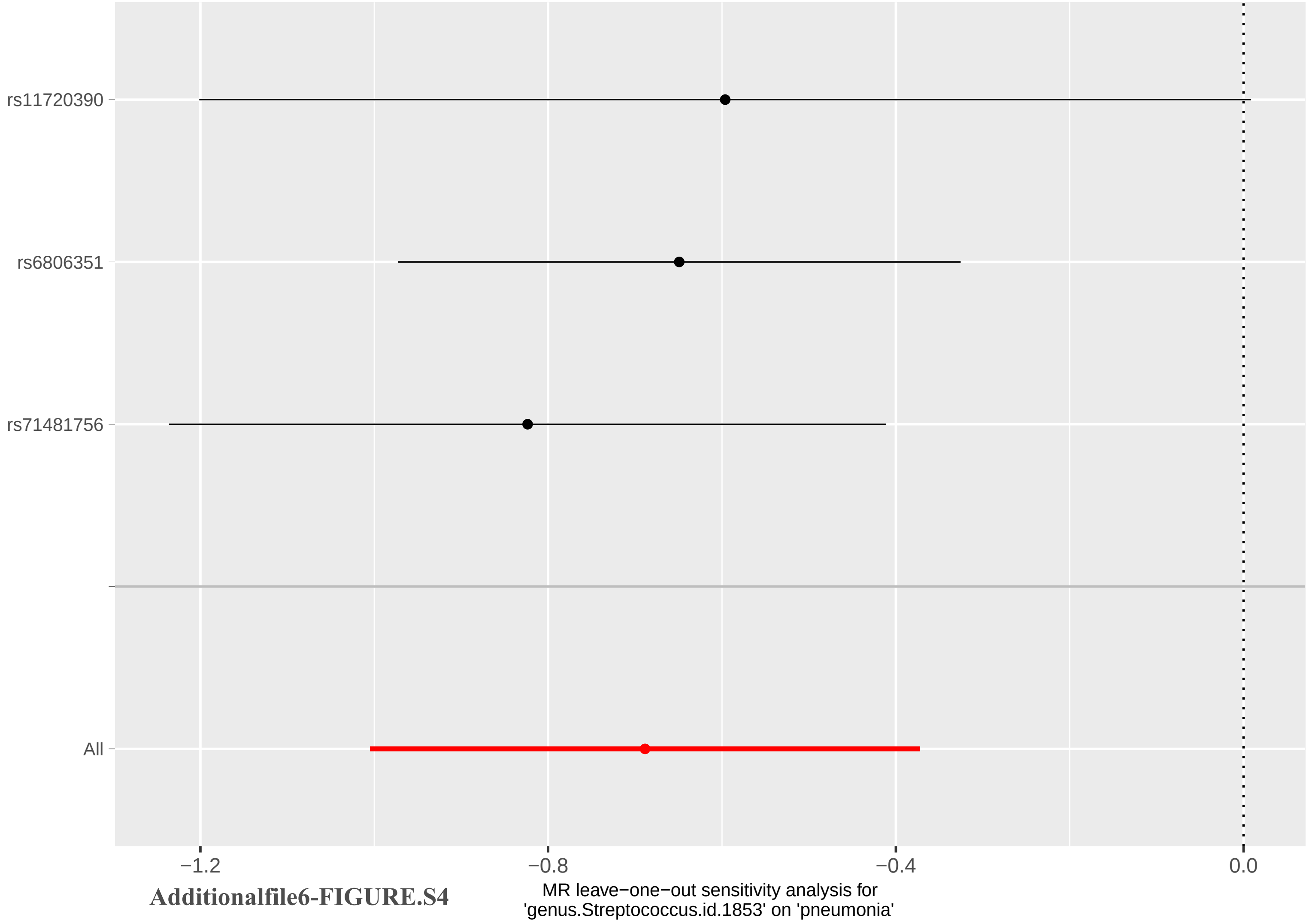

Additionalfile6-FIGURE.S4

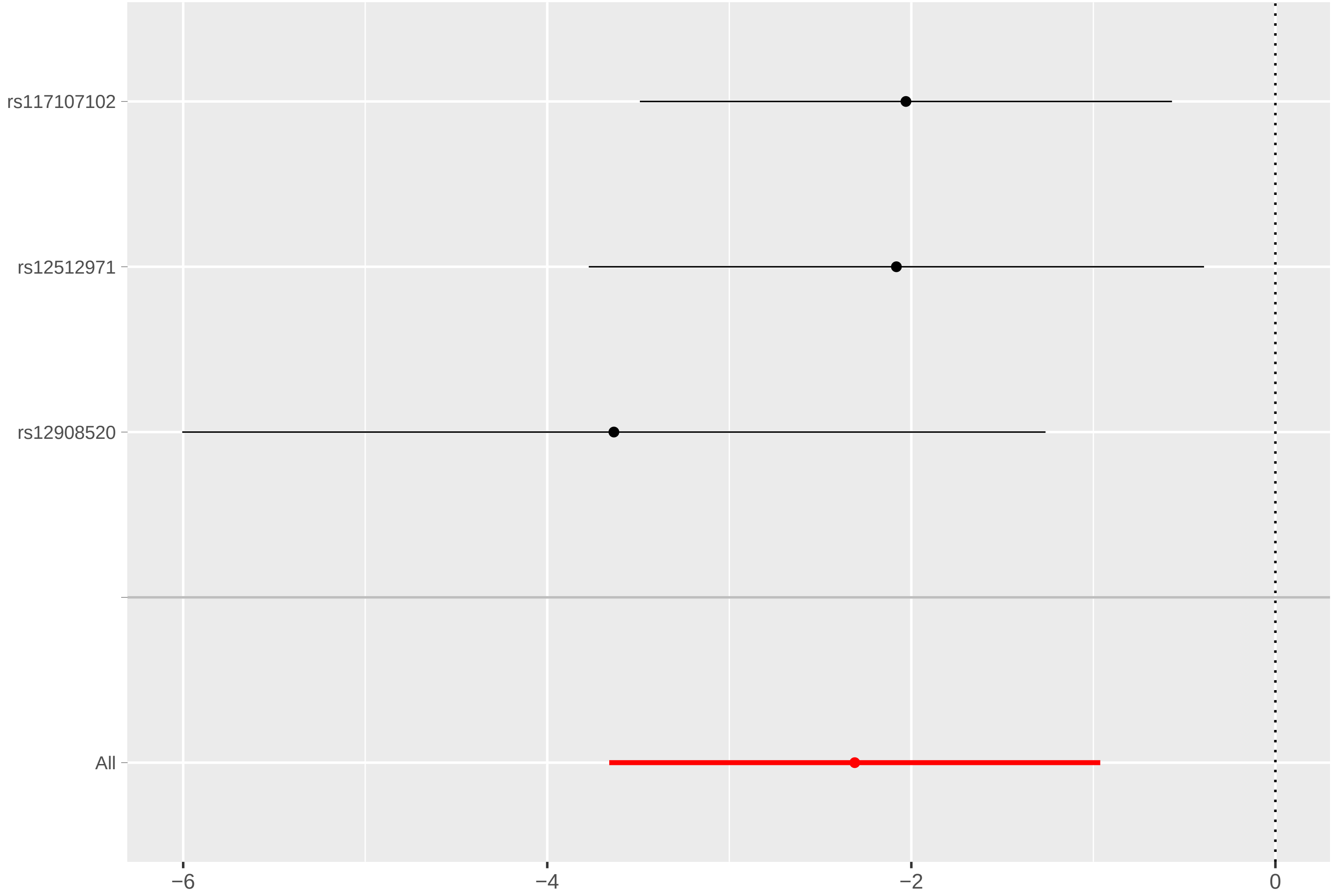

Additionalfile6-FIGURE.S5

MR leave−one−out sensitivity analysis for
'phylum.Verrucomicrobia.id.3982' on 'pneumonia'
