# Supplementary material for: Casual effects of gut microbiota on risk of infections: a two-sample Mendelian randomization study
Source: Front Microbiol. 2023 Oct 10;14:1284723. doi: 10.3389/fmicb.2023.1284723 (PMC10595145; doi:10.3389/fmicb.2023.1284723)
Supplement: Supplementary file 6 [file Data_Sheet_3.PDF]

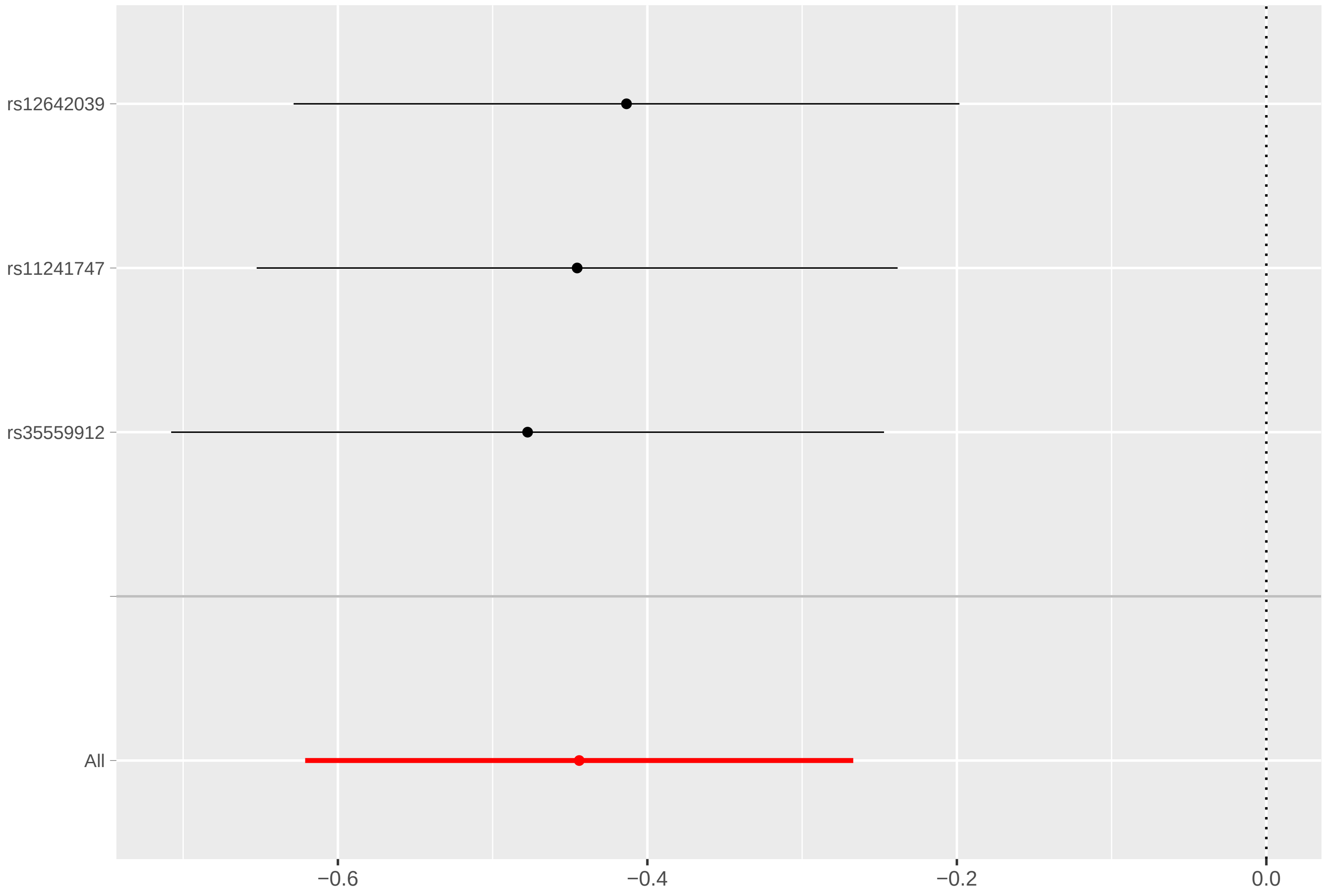

Additionalfile7-FIGURE.S1

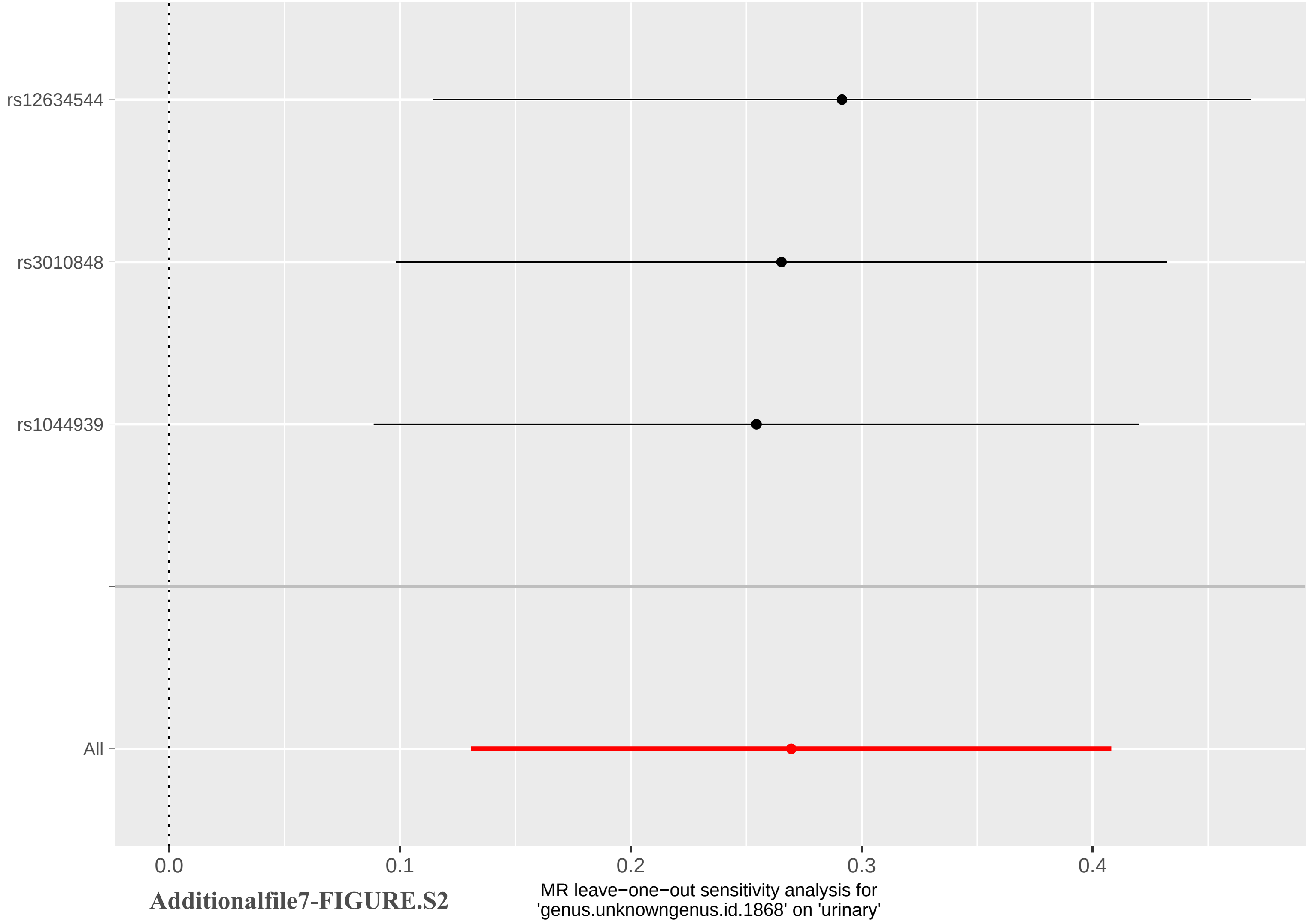

Additionalfile7-FIGURE.S2
